# AGE-RELATED PRESERVATION OF MOTOR NERVE CONDUCTION VELOCITY IN NEURONAL MTORC1 KNOCKDOWN MICE

**DOI:** 10.1093/geroni/igz038.399

**Published:** 2019-11-08

**Authors:** Stacy A Hussong, Veronica Galvan

**Affiliations:** 1 University of Texas Health Science Center at San Antonio, San Antonio, Texas, United States; 2 Barshop Institute for Longevity and Aging Studies, San Antonio, Texas, United States

## Abstract

With age, peripheral nerves undergo demyelination along with overall decrease in peripheral nerve conduction velocity in both sensory and motor nerves. Loss of innervation in muscles is thought to be a major factor in causing age-related sarcopenia including a decrease in muscle function. Dietary restriction attenuates the detrimental effects of aging in mice. Reduction of mTOR signaling is hypothesized to have overlapping mechanisms with dietary restriction. Furthermore, inhibition of mTOR via rapamycin treatment is known to extend lifespan in mice as well as improve peripheral nerve myelination. Therefore, I hypothesized that reducing mTORC1 signaling in neurons would be able to ameliorate the deleterious effects of aging in peripheral nerves. An overall decrease in nerve conduction velocity was observed in both tail sensory and sural nerves with age (15 vs. 30 months). In neuronal mTORC1 KD animals, there was an age-related preservation of both sural and sciatic nerve conduction. Rapamycin treatment produced similar effects with a trend towards increased sciatic nerve conduction velocity in rapamycin-treated wild-type mice at 19 months. The preserve sciatic nerve conduction velocity could be partially explained by preserved myelination. Neuronal mTORC1 knockdown animals had more myelin in the sciatic nerve at 30 mo. as compared to age-matched controls. Overall, these data indicate that mTORC1 signaling plays a role in the age-related decline in peripheral nerve myelination as well as nerve conduction velocity. Future therapeutics could utilize rapamycin or other rapalogs to combat the decline in peripheral nerve function associated with age and other diseases as well.

